# Life expectancy of people with intellectual disability: a retrospective cohort study from New South Wales, Australia

**DOI:** 10.1136/bmjopen-2025-110184

**Published:** 2026-02-04

**Authors:** Preeyaporn Srasuebkul, Julian Trofimovs, Julian Trollor

**Affiliations:** 1 National Centre of Excellence in Intellectual Disability Health, University of New South Wales, Sydney, New South Wales, Australia; 2 Department of Developmental Disability Neuropsychiatry, University of New South Wales, Sydney, New South Wales, Australia

**Keywords:** Health Equity, Mortality, EPIDEMIOLOGY

## Abstract

**ABSTRACT:**

**Objectives:**

To comprehensively analyse the life expectancy at birth of people with intellectual disability, including people with Down syndrome, to inform health equity and service planning.

**Design:**

Retrospective cohort study.

**Setting:**

Residents of New South Wales (NSW), Australia, with intellectual disability from birth onwards between 1 January 2001 and 31 December 2018.

**Participants:**

Our study sample comprised 100 089 individuals with intellectual disability in the dataset, spanning from birth onwards, between 1 January 2001 and 31 December 2018.

**Main outcome measures:**

All cause mortality. Life expectancy was estimated using ordinary and abridged life table methods, based on age-specific population count, and compared with the general NSW population.

**Results:**

Life expectancy at birth of people with intellectual disability was 67 years, 16 years shorter than the NSW average. Males and females with intellectual disability had a similar life expectancy. Individuals with Down syndrome had a life expectancy of 54 years, significantly shorter than those without Down syndrome. The life expectancy gap for people with intellectual disability narrowed with increasing age.

**Conclusion:**

People with intellectual disability in Australia experience a substantial life expectancy gap, with greater disparities for those with Down syndrome. These findings underscore the need for targeted health and social interventions to address systemic inequities and improve health outcomes across the lifespan.

**STRENGTHS AND LIMITATIONS OF THIS STUDY**

- Robust and transparent methodology: The use of formal life table methodology, such as the Chiang method, provides a standardised and widely accepted approach for estimating life expectancy, ensuring transparency and comparability with official population life tables.
- Population-specific baseline data: Our approach directly yields life expectancy estimates for specific populations (intellectual disability, with or without Down syndrome), providing a precise baseline for future calculations of health metrics, including the burden of disease in people with intellectual disability, which cannot be derived from general population life tables.
- Limited confounding adjustment: A key limitation is the method’s inherent inability to directly adjust for multiple confounding variables simultaneously, unlike multivariable regression models.
- Complexity in subgroup comparisons: While separate life tables can be constructed for different subgroups, direct statistical comparison of life expectancy across numerous finely stratified groups becomes complex and less efficient than with a single regression model.
- Data requirements for complete age ranges: Accurate application of complete life table methods is highly dependent on comprehensive and robust age-specific mortality and population data across all age intervals, which can pose challenges, particularly for older ages within specific cohorts.

## Introduction

 Intellectual disability is a neurodevelopmental condition affecting 1–3% of the global population, characterised by significant limitations in intellectual functioning and adaptive behaviour before age 18.^1 2^ Individuals with intellectual disability experience markedly poorer health outcomes, including higher rates of chronic physical and mental health conditions and unmet healthcare needs driven by biological vulnerability, multimorbidity, socioeconomic disadvantage and systemic barriers to equitable healthcare.^3^,^5^ Although life expectancy has improved due to medical advances and community-based care, adults with intellectual disability still live approximately 12 years fewer than the general population.^6^,^8^

Mortality research in this population has traditionally relied on median or average age at death, summary measures that obscure age-specific mortality risks and fail to inform targeted interventions or resource planning. Life expectancy, which is derived from life table methodology, provides a more nuanced understanding of survival patterns across the life course. It enables age-specific analysis, identification of critical periods for intervention and calculation of years of life lost, insights that median or average age at death measures cannot offer.^9^

Recent findings have documented systemic neglect in healthcare provision for people with intellectual disability, evidenced by premature and preventable deaths, inadequate access to quality healthcare and persistent structural barriers within health systems. These investigations have highlighted the urgent need for robust monitoring mechanisms, with life tables serving as essential to enhance accountability and systematically track progress in addressing these profound health inequities.^10^

The impact of these barriers is exemplified by health outcomes among people with Down syndrome, the most common genetic cause of intellectual disability, who face increased risks of congenital heart defects, early-onset dementia and autoimmune conditions.^11^ While life expectancy has risen dramatically from 25 years in 1983 to nearly 60 years recently, substantial disparities persist compared with both the general population and people with intellectual disability without Down syndrome.^7 12 13^ Racial and ethnic minorities experience less pronounced survival improvements, and condition-specific risks continue to drive elevated mortality rates.^14 15^

While life table methods have been used internationally to estimate life expectancy in this population, no comprehensive analysis exists using Australian data. This study addresses this gap using linked administrative datasets from New South Wales (NSW) to estimate life expectancy for people with intellectual disability, including those with and without Down syndrome. This represents the first life table analysis of its kind in Australia, providing robust, policy-relevant estimates and highlighting the persistent disparities in the Australian context.^16^,^19^

## Methods

### Data sources and cohort definition

We sourced data from a linked population-based data asset. This asset contains health and service records of people with intellectual disability and matched comparators without intellectual disability in NSW, Australia.^20^ The linked data set comprised 20 individual administrative data sets from health, disability and other services. Nine of these datasets were available for cohort identification at the time of the study: Disability Service Minimum Dataset (DSMDS); Admitted Patient Data Collection; Mental Health Ambulatory Data; Emergency Department Data Collection; NSW Department of Education (students who received targeted specialist support in public schools; Statewide Disability Services, Corrective Services NSW; NSW Public Guardian and NSW Ombudsman. These nine datasets contained specific diagnosis codes or service provision flags necessary to identify individuals with intellectual disability.

An individual was included in our study cohort if they were identified through at least one of the following pathways: (1) appearing in a disability service dataset with intellectual disability recorded as their primary or secondary disability and (2) appearing in a health administrative dataset with a recorded diagnosis of intellectual disability.

For this study, ‘appearing in a disability service dataset’ (eg, DSMDS) was defined as accessing one or more state-funded supports such as supported accommodation, community participation or respite care.

The remaining 11 datasets (eg, the NSW Registry of Births, Deaths and Marriages (RBDM) or Cancer Registry) were not used to identify individuals with intellectual disability as they do not contain these specific identifiers. Instead, these datasets were used to ascertain outcomes (ie, mortality or cancer incidence) and establish covariates. Specifically, date of death information from the NSW RBDM and the National Death Index (NDI) datasets was used to determine the end of follow-up.

Our study sample comprised 100 089 individuals with intellectual disability in the dataset, spanning from birth onwards, between 1 January 2001 and 31 December 2018. We excluded individuals who had died before 1 January 2001 and those born after 31 December 2018. The sample was stratified by sex and Down syndrome status. We identified a subpopulation of individuals with Down syndrome using diagnostic codes from the linked datasets (International Classification of Diseases (ICD)-9 code 758.0, ICD-10 code Q90 or ICD-10-AM code U88.2) or any flag of Down syndrome from disability service datasets.

Individuals entered the study cohort on 1 January 2001 or their date of birth, whichever occurred later. Follow-up for each individual continued from this entry date until their date of death (ascertained via linkage to the NSW RBDM and the NDI) or the end of the study period (31 December 2018), whichever occurred first. As our outcome (mortality) was ascertained through comprehensive registry linkage, there was no loss to follow-up.

Regarding missing data, variables essential for life table construction (date of birth, sex and date of death) were requisite for inclusion and were virtually complete. For the Down syndrome stratification, individuals were classified based on specific diagnostic codes or service flags; those without such a record were categorised as ‘intellectual disability without Down syndrome’.

### Life expectancy estimation

Life expectancy refers to the predicted average number of additional years an individual is expected to live, given current age-specific and sex-specific mortality rates. We estimated life expectancy at birth for individuals with intellectual disability. These estimates were stratified by sex and Down syndrome status.

We constructed complete period life tables for our study cohort, using 1-year age intervals, for all individuals. This involves calculating the probability of dying within each age interval from the observed age-specific death rates within our cohort. From these probabilities, we then determined the number of survivors at each age and the total person-years lived.

These cohort-specific estimates were then compared with the general NSW population using standard life table methods based on publicly available data from the Australian Bureau of Statistics.^21^

Where small cell sizes necessitated aggregation, we generated abridged life tables for grouped age ranges (eg, 5-year intervals). For these abridged tables, we used standard actuarial techniques to estimate mortality probabilities from the grouped age data.^22 23^ Abridged life tables typically produce slightly lower life expectancy estimates due to methodological differences in age grouping and interpolation methods.

Finally, we derived life expectancy at birth and remaining life expectancy at selected ages (eg, 20, 40, 60) directly from these calculations. Given that the latest birth year included in our cohort was 2018, life expectancy at birth derived from these period life tables can be interpreted as the life expectancy for individuals with intellectual disability who were born in 2018, assuming they were subject to the mortality rates observed during the study period for their entire lives. We calculated 95% CIs for life expectancy at birth using the parametric bootstrap method, assuming a Poisson distribution of deaths in each group with 1000 replications.

To visually represent the disparities and trends in life expectancy across the lifespan, we generated figures illustrating the difference in remaining life expectancy. The plot shows the life expectancy of individuals with intellectual disability minus that of the general NSW population across different ages, stratified by sex. All analyses were performed using Stata V.18.0 (StataCorp, College Station, TX, USA).

## Results

Detailed cohort characteristics have been reported previously.^20^ Briefly, the study included 100 089 individuals with intellectual disability, of whom 34% were female, and a median age at cohort entry of 3 years (IQR, 0–19). Down syndrome was present in 4% of the cohort, and 8911 deaths occurred during the study period.

Based on our life table (table 1), the life expectancy at birth for individuals with intellectual disability born in 2018 was 67.03 years (95% CI 60.0 to 73.6 years). This represents a substantial disparity compared with the 83.3 years of life expectancy at birth in the general NSW population born between 2018 and 2020.^24^

**Table 1:** Life table, individuals with intellectual disability, NSW, 2001–2019

Age	Number alive	Mortality rate	Number of person-years lived	Life expectancy
0	100 000	0.00601	99 700	67.02669
1	99 401	0.00187	99 308	66.42740
2	99 215	0.00095	99 168	65.55109
3	99 121	0.00065	99 088	64.61318
4	99 056	0.00071	99 021	63.65494
5	98 985	0.00090	98 941	62.70007
6	98 896	0.00046	98 873	61.75626
7	98 850	0.00069	98 817	60.78444
8	98 783	0.00080	98 743	59.82584
9	98 704	0.00070	98 669	58.87321
10	98 635	0.00076	98 597	57.91407
11	98 559	0.00069	98 525	56.95794
12	98 491	0.00073	98 455	55.99707
13	98 419	0.00107	98 367	55.03768
14	98 314	0.00096	98 267	54.09579
15	98 220	0.00107	98 168	53.14729
16	98 115	0.00084	98 074	52.20339
17	98 033	0.00127	97 971	51.24693
18	97 908	0.00145	97 837	50.31147
19	97 766	0.00202	97 668	49.38390
20	97 569	0.00213	97 465	48.48251
21	97 362	0.00203	97 263	47.58498
22	97 164	0.00211	97 062	46.68056
23	96 960	0.00205	96 861	45.77794
24	96 761	0.00277	96 628	44.87085
25	96 494	0.00278	96 360	43.99380
26	96 226	0.00278	96 092	43.11496
27	95 959	0.00335	95 798	42.23354
28	95 638	0.00332	95 479	41.37356
29	95 321	0.00276	95 190	40.50949
30	95 059	0.00399	94 869	39.61990
31	94 680	0.00395	94 493	38.77646
32	94 306	0.00429	94 105	37.92796
33	93 903	0.00450	93 692	37.08875
34	93 481	0.00423	93 284	36.25393
35	93 087	0.00572	92 821	35.40541
36	92 556	0.00546	92 304	34.60548
37	92 052	0.00378	91 878	33.79235
38	91 704	0.00413	91 515	32.91845
39	91 326	0.00522	91 088	32.05269
40	90 850	0.00526	90 612	31.21799
41	90 374	0.00488	90 153	30.38006
42	89 934	0.00604	89 663	29.52617
43	89 392	0.00774	89 047	28.70203
44	88 703	0.00700	88 393	27.92127
45	88 084	0.00800	87 733	27.11378
46	87 382	0.00939	86 973	26.32761
47	86 566	0.00957	86 153	25.57115
48	85 742	0.00887	85 363	24.81219
49	84 985	0.01138	84 503	24.02877
50	84 023	0.00982	83 612	23.29800
51	83 202	0.01167	82 718	22.52300
52	82 237	0.01069	81 799	21.78152
53	81 363	0.01605	80 713	21.01020
54	80 067	0.01803	79 350	20.34203
55	78 637	0.01568	78 023	19.70300
56	77 413	0.01931	76 671	19.00650
57	75 933	0.01746	75 274	18.36743
58	74 618	0.02331	73 755	17.68214
59	72 899	0.02416	72 025	17.08749
60	71 158	0.02273	70 356	16.49323
61	69 559	0.02847	68 578	15.86097
62	67 607	0.02758	66 683	15.30471
63	65 767	0.03133	64 748	14.71880
64	63 739	0.03390	62 670	14.17143
65	61 614	0.03256	60 622	13.64295
66	59 640	0.04016	58 458	13.07807
67	57 292	0.04251	56 092	12.59366
68	54 908	0.04205	53 769	12.11895
69	52 647	0.04870	51 386	11.61811
70	50 145	0.05107	48 886	11.17311
71	47 648	0.05196	46 431	10.73256
72	45 235	0.06170	43 868	10.27854
73	42 529	0.05771	41 325	9.90122
74	40 144	0.06209	38 923	9.45999
75	37 727	0.06657	36 499	9.03425
76	35 298	0.07603	33 989	8.62208
77	32 714	0.06490	31 675	8.26415
78	30 658	0.07817	29 490	7.78514
79	28 353	0.10530	26 911	7.37799
80	25 519	0.09439	24 351	7.14276
81	23 220	0.09859	22 112	6.80109
82	21 040	0.10508	19 972	6.45483
83	18 941	0.11009	17 936	6.11560
84	16 967	0.11298	16 043	5.77022
85	15 154	0.13380	14 184	5.40169
86	13 256	0.15094	12 304	5.10505
87	11 399	0.16834	10 491	4.85741
88	9633	0.15839	8909	4.65887
89	8222	0.14760	7644	4.37487
90	7094	0.17273	6515	3.99312
91	5968	0.19048	5434	3.65443
92	4933	0.13077	4624	3.31964
93	4329	0.27778	3779	2.71508
94	3279	0.12346	3084	2.43174
95	2898	0.23077	2588	1.68692
96	2301	1	2301	1

NSW, New South Wales.

For females with intellectual disability born in 2018, life expectancy at birth was 66.81 years (95% CI 57.1 to 76.3 years), compared with 85.3 years for the general NSW female population.^25^ This represented a stark reduction of 18.49 years for females with intellectual disability at birth. Remaining life expectancy was 49.12 years at age 20 and 17.32 years at age 60 (table 2).

**Table 2:** Life table, individuals with intellectual disability by sex, New South Wales, 2001–2019

Age	Female				Male			
	Number alive	Mortality rate	Number of person-years lived	Life expectancy	Number alive	Mortality rate	Number of person-years lived	Life expectancy
0	100 000	0.01080	99 459	66.81006	100 000	0.00418	99 791	66.94013
1	98 920	0.00328	98 757	66.53429	99 582	0.00135	99 514	66.21928
2	98 595	0.00159	98 517	65.75145	99 447	0.00071	99 412	65.30808
3	98 438	0.00142	98 368	64.85551	99 376	0.00036	99 358	64.35444
4	98 299	0.00127	98 236	63.94706	99 340	0.00050	99 315	63.37764
5	98 173	0.00208	98 071	63.02790	99 290	0.00046	99 267	62.40937
6	97 969	0.00039	97 950	62.15833	99 245	0.00049	99 220	61.43768
7	97 931	0.00118	97 874	61.18214	99 196	0.00050	99 171	60.46740
8	97 816	0.00115	97 760	60.25367	99 147	0.00066	99 114	59.49731
9	97 703	0.00097	97 656	59.32269	99 081	0.00059	99 052	58.53625
10	97 609	0.00103	97 558	58.37968	99 022	0.00066	98 990	57.57075
11	97 508	0.00110	97 454	57.43950	98 958	0.00052	98 932	56.60820
12	97 400	0.00112	97 345	56.50244	98 906	0.00057	98 877	55.63767
13	97 291	0.00155	97 215	55.56549	98 849	0.00086	98 807	54.66903
14	97 140	0.00154	97 065	54.65078	98 764	0.00071	98 729	53.71580
15	96 990	0.00155	96 915	53.73434	98 694	0.00085	98 652	52.75376
16	96 840	0.00140	96 772	52.81696	98 609	0.00059	98 580	51.79845
17	96 704	0.00156	96 629	51.89050	98 551	0.00114	98 495	50.82880
18	96 554	0.00107	96 502	50.97073	98 439	0.00163	98 358	49.88616
19	96 451	0.00192	96 358	50.02461	98 278	0.00206	98 177	48.96703
20	96 266	0.00241	96 150	49.11973	98 075	0.00199	97 978	48.06710
21	96 034	0.00258	95 910	48.23703	97 880	0.00173	97 795	47.16195
22	95 786	0.00273	95 656	47.36038	97 711	0.00176	97 625	46.24297
23	95 525	0.00246	95 407	46.48868	97 539	0.00181	97 451	45.32341
24	95 290	0.00214	95 188	45.60199	97 363	0.00314	97 210	44.40471
25	95 086	0.00278	94 954	44.69882	97 057	0.00278	96 922	43.54279
26	94 822	0.00298	94 680	43.82200	96 788	0.00264	96 660	42.66266
27	94 539	0.00302	94 396	42.95153	96 532	0.00356	96 360	41.77429
28	94 253	0.00329	94 098	42.08022	96 189	0.00333	96 028	40.92172
29	93 943	0.00233	93 833	41.21766	95 868	0.00305	95 722	40.05671
30	93 724	0.00400	93 536	40.31287	95 576	0.00398	95 386	39.17754
31	93 349	0.00330	93 195	39.47270	95 196	0.00440	94 987	38.33197
32	93 041	0.00520	92 799	38.60181	94 777	0.00360	94 607	37.49934
33	92 557	0.00448	92 349	37.80098	94 436	0.00450	94 223	36.63286
34	92 142	0.00386	91 964	36.96886	94 011	0.00449	93 800	35.79634
35	91 786	0.00554	91 532	36.11018	93 589	0.00582	93 316	34.95551
36	91 278	0.00509	91 046	35.30847	93 044	0.00572	92 778	34.15726
37	90 813	0.00408	90 628	34.48665	92 512	0.00354	92 348	33.35098
38	90 443	0.00372	90 275	33.62579	92 184	0.00443	91 980	32.46774
39	90 107	0.00541	89 862	32.74951	91 775	0.00505	91 543	31.61009
40	89 619	0.00524	89 384	31.92509	91 312	0.00526	91 071	30.76812
41	89 149	0.00640	88 864	31.09055	90 831	0.00367	90 665	29.92808
42	88 579	0.00681	88 277	30.28750	90 498	0.00541	90 253	29.03650
43	87 976	0.00698	87 669	29.49168	90 008	0.00829	89 634	28.19185
44	87 362	0.00703	87 054	28.69544	89 262	0.00692	88 953	27.42335
45	86 748	0.00761	86 417	27.89513	88 644	0.00826	88 277	26.61106
46	86 087	0.01034	85 641	27.10534	87 912	0.00853	87 537	25.82842
47	85 197	0.00916	84 806	26.38334	87 162	0.00982	86 733	25.04638
48	84 417	0.00732	84 108	25.62251	86 306	0.01007	85 871	24.28985
49	83 799	0.01064	83 352	24.80777	85 437	0.01187	84 929	23.53181
50	82 907	0.00842	82 558	24.06918	84 423	0.01088	83 963	22.80838
51	82 210	0.01168	81 729	23.26923	83 505	0.01154	83 022	22.05373
52	81 249	0.01199	80 761	22.53831	82 541	0.00951	82 148	21.30534
53	80 275	0.01498	79 672	21.80585	81 756	0.01669	81 072	20.50511
54	79 072	0.01829	78 347	21.12998	80 392	0.01752	79 686	19.84470
55	77 626	0.01720	76 957	20.51427	78 984	0.01418	78 422	19.18960
56	76 291	0.01770	75 613	19.86467	77 864	0.02033	77 070	18.45847
57	74 940	0.01700	74 301	19.21368	76 281	0.01757	75 609	17.83113
58	73 666	0.02323	72 807	18.53725	74 941	0.02289	74 080	17.14113
59	71 955	0.02268	71 136	17.96634	73 225	0.02489	72 310	16.53095
60	70 323	0.02302	69 510	17.37170	71 403	0.02201	70 614	15.94022
61	68 704	0.02671	67 783	16.76931	69 831	0.02923	68 806	15.28775
62	66 869	0.02574	66 005	16.21578	67 790	0.02844	66 821	14.73313
63	65 148	0.03379	64 041	15.63107	65 862	0.02838	64 923	14.14987
64	62 947	0.02986	62 002	15.16029	63 993	0.03619	62 828	13.54857
65	61 067	0.03029	60 138	14.61159	61 677	0.03350	60 638	13.03861
66	59 218	0.03578	58 152	14.05244	59 611	0.04233	58 340	12.47333
67	57 099	0.03917	55 973	13.55547	57 088	0.04364	55 833	12.00271
68	54 862	0.03813	53 809	13.08788	54 596	0.04374	53 393	11.52781
69	52 770	0.05177	51 392	12.58695	52 208	0.04390	51 054	11.03237
70	50 039	0.03988	49 034	12.24705	49 917	0.05813	48 451	10.51609
71	48 043	0.05078	46 813	11.73513	47 015	0.05050	45 817	10.13459
72	45 603	0.06178	44 180	11.33646	44 641	0.05810	43 331	9.64725
73	42 786	0.04837	41 743	11.05031	42 047	0.06286	40 711	9.21175
74	40 716	0.05296	39 629	10.58679	39 404	0.06656	38 077	8.79649
75	38 560	0.05661	37 458	10.15106	36 781	0.07136	35 453	8.38850
76	36 378	0.06863	35 114	9.73045	34 156	0.07735	32 818	7.99520
77	33 881	0.05043	33 019	9.41102	31 514	0.07448	30 326	7.62415
78	32 172	0.05991	31 199	8.88451	29 167	0.08965	27 839	7.19794
79	30 245	0.09684	28 756	8.41918	26 552	0.10322	25 157	6.85829
80	27 316	0.07453	26 285	8.26918	23 812	0.10644	22 521	6.59117
81	25 280	0.07283	24 348	7.89536	21 277	0.11727	20 004	6.31789
82	23 439	0.08531	22 424	7.47673	18 782	0.11666	17 664	6.09214
83	21 440	0.08729	20 490	7.12814	16 591	0.12535	15 528	5.83208
84	19 568	0.08076	18 767	6.76275	14 511	0.14164	13 457	5.59782
85	17 988	0.11392	16 943	6.31360	12 456	0.14173	11 551	5.44110
86	15 939	0.12503	14 920	6.06230	10 690	0.16337	9791	5.25916
87	13 946	0.13964	12 948	5.85870	8944	0.17911	8117	5.19141
88	11 998	0.13801	11 150	5.73048	7342	0.16054	6735	5.21863
89	10 343	0.11287	9747	5.56992	6163	0.17495	5607	5.12384
90	9175	1	47 860	5.21622	5085	1	25 973	5.10769

Males with intellectual disability born in 2018 had a life expectancy at birth of 66.94 years (95% CI 56.7 to 77.0 years), 14.26 years shorter than their peers in the general NSW male population. The life expectancy at birth for males in the general NSW population (2018–2020) was 81.2 years. Their remaining life expectancy was 49.18 years at age 20, and 16.54 years for those aged 60 (table 2).

Across all age cohorts, males and females with intellectual disability showed very similar life expectancies at birth, with females having a slightly lower life expectancy at birth. However, both sexes experienced significantly lower life expectancy at birth compared with their respective sex in the general NSW population.

**Figure 1:**
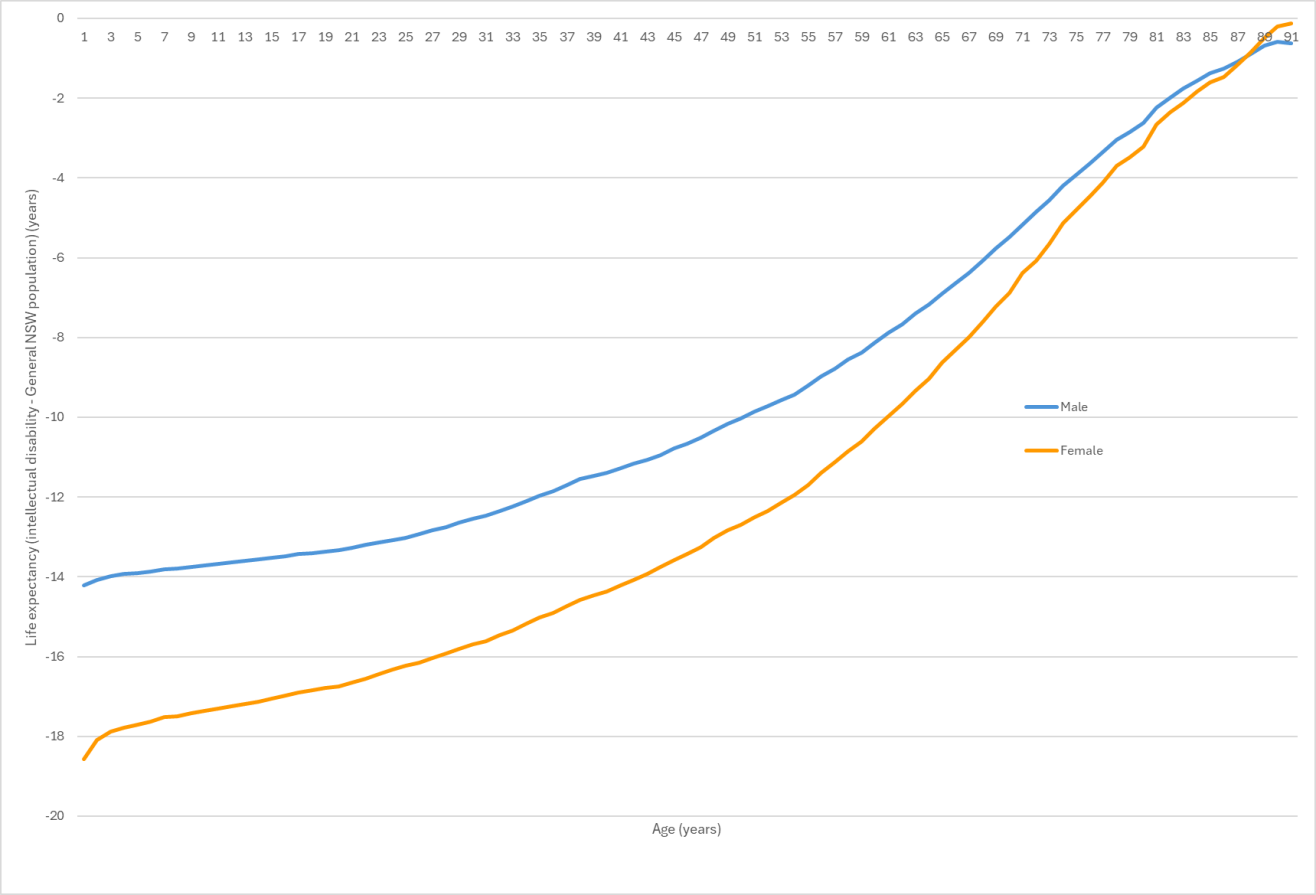
Life expectancy deficit for individuals with intellectual disability (compared with the general NSW population). NSW, New South Wales.

While a substantial gap in life expectancy exists at birth compared with the general NSW population, this disparity tends to diminish with age for both sexes. For example, at age 65 years, the remaining life expectancy for females with intellectual disability was 14.61 years. When compared with females in the general NSW population at the same age, whose remaining life expectancy was typically 22.91 years, the initial broad gap seen at birth was reduced to 8.30 years. Similarly, for males with intellectual disability, remaining life expectancy at age 65 years was 13.04 years. Compared with males in the general NSW population at the same age, whose remaining life expectancy was typically 20.27 years, this initial gap was reduced to 6.62 years. This trend is further illustrated in figure 1, which plots the difference in life expectancy between individuals with intellectual disability and the general NSW population across the age spectrum, highlighting the reduction in disparity at older ages, stratified by sex.

Table 3 presents abridged life tables for individuals with intellectual disability stratified by Down syndrome status. For individuals with intellectual disability but without Down syndrome born in 2018, life expectancy at birth was 66.31 years (95% CI 45.4 to 91.0 years). Their remaining life expectancy was 47.60 years at age 20 and 11.28 years at age 65.

**Table 3:** Life table, individuals with intellectual disability by Down syndrome, New South Wales, 2001–2019

Age	Without Down syndrome				With Down syndrome			
	Number alive	Mortality rate	Number of person-years lived	Life expectancy	Number alive	Mortality rate	Number of person-years lived	Life expectancy
0	100 000	0.00479	99 760	66.30850	100 000	0.03713	98 132	54.55145
1	99 521	0.00359	397 367	65.62552	96 287	0.01784	381 701	55.63616
5	99 163	0.00348	494 954	61.85476	94 569	0.00553	471 538	52.61034
10	98 818	0.00403	493 095	57.06191	94 047	0.00921	468 065	47.88879
15	98 420	0.00640	490 523	52.28282	93 181	0.00755	464 143	43.31060
20	97 790	0.01080	486 306	47.60350	92 477	0.01376	459 198	38.62102
25	96 734	0.01463	480 124	43.09594	91 205	0.01839	451 818	34.12505
30	95 319	0.02045	471 705	38.69868	89 527	0.02486	442 050	29.71774
35	93 370	0.02328	461 393	34.45454	87 302	0.03484	428 860	25.41188
40	91 196	0.02916	449 299	30.21646	84 260	0.04597	411 541	21.23947
45	88 537	0.04396	432 880	26.04933	80 386	0.06999	387 696	17.14345
50	84 644	0.05531	411 407	22.13309	74 760	0.15639	343 742	13.24779
55	79 963	0.08099	383 397	18.28392	63 068	0.25658	272 889	10.25341
60	73 487	0.11584	345 717	14.67796	46 886	0.38486	185 679	7.97197
65	64 975	0.16690	296 938	11.28013	28 841	0.52431	101 763	6.52172
70	54 130	0.23912	236 820	8.05434	13 719	0.47387	50 616	6.29269
75	41 187	0.31820	171 083	4.83566	7218	0.28888	30 583	4.94798
80	28 081	1	28 081	1	5133	1	5133	1

Individuals with intellectual disability and Down syndrome born in 2018 had a considerably lower life expectancy at birth of 54.55 years (95% CI 34.6 to 75.1 years). Their remaining life expectancy was 38.62 years at age 20. This represents an 11.76-year reduction in life expectancy at birth for individuals with Down syndrome compared with those with intellectual disability without Down syndrome.

## DISCUSSION

This study provides contemporary and robust estimates of life expectancy for individuals with intellectual disability in NSW, Australia. We found a substantial 16-year reduction in life expectancy at birth (67 years) compared with the general NSW population, with particularly pronounced disparities for individuals with Down syndrome (54.55 years). While males and females with intellectual disability had similar life expectancy, the disparity with the general population was greater for females.^26^ The life expectancy gap diminished with age across all subgroups.

Our findings align with international literature from high-income countries reporting a 13–20 year reduction in life expectancy for people with intellectual disability. Studies from the UK, the USA and Canada have documented similar disparities, with some reporting gaps for up to 19.7 years at birth.^14 27^ This consistency across different healthcare systems and countries suggests our NSW findings may be generalisable to similar high-income settings with universal healthcare. The particularly stark reduction for Down syndrome reflects well-documented health vulnerabilities associated with this condition globally.^12^ Despite improvements over recent decades, significant disparities persist.^28 29^

The observed life expectancy for people with intellectual disability in NSW was comparable to the general population’s life expectancy in many low-income and middle-income countries, despite Australia’s universal healthcare system.^30^ This reflects systemic challenges in accessing appropriate healthcare and has been described as ‘systemic neglect’.^4 31^ Contributing factors include higher rates of avoidable deaths from treatable conditions, with respiratory and circulatory diseases being the leading causes of premature mortality.^432^,^35^

These findings underscore the urgent need for comprehensive implementation of the National Roadmap for Improving the Health of People with Intellectual Disability, as well as the health-related recommendations from the Disability Royal Commission.^36^ For clinicians, the data highlight the need for enhanced training, proactive health surveillance and person-centred care models addressing the complex needs of individuals with intellectual disability across their lifespan. Social determinants of health also play a role, indicating the need for a multifaceted approach addressing both healthcare access and broader social factors.^10^

The convergence of life expectancy differences at older ages suggests that excess mortality is concentrated in earlier life, indicating opportunities for targeted interventions during critical periods. However, findings should be interpreted cautiously given the limitations of administrative data and our descriptive approach. While we cannot establish causal mechanisms, the magnitude and consistency of disparities across age groups suggest systematic factors beyond individual health conditions contribute to excess mortality, requiring comprehensive health system responses addressing both medical care and social determinants of health.

### Limitations

This study has several notable strengths. We used a large population-based linked administrative dataset from NSW, Australia’s most populous state representing approximately one-third of the national population, providing robust and contemporary estimates. The application of formal life table methodology ensured transparency and direct comparable estimates. Our approach enabled detailed stratification by sex and Down syndrome status, yielding population-specific life expectancy estimates crucial for health planning and policy development.

However, a primary limitation is our reliance on administrative data for cohort formation. We acknowledge that our sample is an administrative cohort and not a complete population sample, as it does not capture the entire population of people with intellectual disability in NSW. This methodology likely underestimates the intellectual disability population, as individuals not accessing services, and those with intellectual disability who are not identified as such are excluded. The direction of this bias is uncertain: excluded individuals may have died before accessing services (leading to overestimated life expectancy) or may have mortality rates similar to the general population if their disability level does not require specialised services (leading to underestimated life expectancy disparities). The proportion of the intellectual disability population captured by administrative data is unknown, making it difficult to assess the magnitude and direction of potential bias.

Despite this limitation in generalisability, the use of life tables remains the gold standard and most statistically robust method for calculating and presenting age-specific mortality rates and life expectancy for any defined longitudinal cohort. A core objective of this study was to comprehensively analyse life expectancy for people with intellectual disability to inform health equity and service planning. This objective required the generation of robust, cohort-specific estimates, as applying general population tables would be inappropriate and misrepresent the true mortality burden for this population.

Our descriptive approach cannot establish causality or directly adjust for confounding factors such as socioeconomic status, comorbidities and healthcare access. The direction of residual confounding is unclear—while people with intellectual disability face socioeconomic disadvantages and increased mortality risk, they may receive enhanced healthcare monitoring that could be protective.

Geographic limitation to NSW limits external validity, as findings may not generalise to other Australian states with different population demographics or service models. Variations in disability services, healthcare access and socioeconomic contexts across countries further limit international generalisability. Period life tables reflect 2014–2018 mortality patterns rather than true cohort survival, and our general population comparison uses aggregate rather than individually matched data.

## Conclusion

This study demonstrates significant life expectancy disparities for individuals with intellectual disability in NSW, with a 16-year reduction at birth and additional 12-year reduction for Down syndrome. These findings underscore persistent health inequities in a high-income country with universal healthcare and emphasise the urgent need for targeted interventions and healthcare system reforms to ensure equitable access to quality care throughout the lifespan.

## Data Availability

No data are available.

## References

[1] Schalock RL, Borthwick-Duffy SA, Bradley VJ (2010). Intellectual Disability: Definition, Classification, and Systems of Supports.

[2] Maulik PK, Mascarenhas MN, Mathers CD (2011). Prevalence of intellectual disability: a meta-analysis of population-based studies. Res Dev Disabil.

[3] Hosking FJ, Carey IM, Shah SM (2016). Mortality Among Adults With Intellectual Disability in England: Comparisons With the General Population. Am J Public Health.

[4] Trollor J, Srasuebkul P, Xu H (2017). Cause of death and potentially avoidable deaths in Australian adults with intellectual disability using retrospective linked data. BMJ Open.

[5] Emerson E, Hatton C (2008). Socioeconomic disadvantage, social participation and networks and the self-rated health of English men and women with mild and moderate intellectual disabilities: cross sectional survey. Eur J Public Health.

[6] Landes SD, Stevens JD, Turk MA (2021). Cause of death in adults with intellectual disability in the United States. J Intellect Disabil Res.

[7] Bittles AH, Glasson EJ (2004). Clinical, social, and ethical implications of changing life expectancy in Down syndrome. Dev Med Child Neurol.

[8] Lauer E, McCallion P (2015). Mortality of People with Intellectual and Developmental Disabilities from Select US State Disability Service Systems and Medical Claims Data. J Appl Res Intellect Disabil.

[9] Tyrer F, McGrother C (2009). Cause-specific mortality and death certificate reporting in adults with moderate to profound intellectual disability. J Intellect Disabil Res.

[10] Tyrer F, Morriss R, Kiani R (2022). Mortality disparities and deprivation among people with intellectual disabilities in England: 2000-2019. J Epidemiol Community Health.

[11] Roizen NJ, Patterson D (2003). Down’s syndrome. Lancet.

[12] Coppus AMW (2013). People with intellectual disability: what do we know about adulthood and life expectancy?. Dev Disabil Res Rev.

[13] Glasson EJ, Sullivan SG, Hussain R (2003). Comparative survival advantage of males with Down syndrome. Am J Hum Biol.

[14] Glover G, Williams R, Heslop P (2017). Mortality in people with intellectual disabilities in England. J Intellect Disabil Res.

[15] Landes SD, Turk MA, Wong A (2021). COVID-19 outcomes among people with intellectual and developmental disability in California: The importance of type of residence and skilled nursing care needs. Disabil Health J.

[16] Patja K, Iivanainen M, Vesala H (2000). Life expectancy of people with intellectual disability: a 35-year follow-up study. J Intellect Disabil Res.

[17] Baird PA, Sadovnick AD (1989). Life tables for Down syndrome. Hum Genet.

[18] Day SM, Strauss DJ, Shavelle RM (2005). Mortality and causes of death in persons with Down syndrome in California. Dev Med Child Neurol.

[19] Rotenberg S, Kuper H (2025). Left behind: modelling the life expectancy disparities amongst people with disabilities in low and middle-income countries. medRxiv.

[20] Reppermund S, Srasuebkul P, Vajdic CM (2024). Cohort profile: understanding health service system needs for people with intellectual disability using linked data in New South Wales, Australia. Epidemiol Health.

[21] Australian Bureau of Statistics (2025). Life expectancy methodology, 2021–2023.

[22] CHIANG CL (1960). A stochastic study of the life table and its applications. II. Sample variance of the observed expectation of life and other biometric functions. Hum Biol.

[23] Chiang CL (1984). Life Table and Its Applications.

[24] Australian Bureau of Statistics (2022). Life tables, 2019–2021.

[25] Australian Bureau of Statistics (2021). Life tables, 2018–2020.

[26] Robertson J, Heslop P, Lauer E (2021). Gender and the Premature Deaths of People with Intellectual Disabilities: An International Expert Consultation. Policy Practice Intel Disabi.

[27] McCarthy J, O’Hara J (2011). Ill-health and intellectual disabilities. Curr Opin Psychiatry.

[28] Dolan E, Lane J, Hillis G (2021). Changing trends in life expectancy in intellectual disability over time.

[29] Landes SD, McDonald KE, Wilmoth JM (2021). Evidence of continued reduction in the age-at-death disparity between adults with and without intellectual and/or developmental disabilities. J Appl Res Intellect Disabil.

[30] United Nations (2025). World population prospects.

[31] Scheepers M, Kerr M, O’Hara D (2005). Reducing Health Disparity in People with Intellectual Disabilities: A Report from Health Issues Special Interest Research Group of the International Association for the Scientific Study of Intellectual Disabilities^1^. Policy Practice Intel Disabi.

[32] Tyrer F, Morriss R, Kiani R (2022). Health Needs and Their Relationship with Life Expectancy in People with and without Intellectual Disabilities in England. Int J Environ Res Public Health.

[33] Thygesen LC, Klitgaard MB, Sabers A (2024). Potentially avoidable mortality among adults with intellectual disability. Eur J Public Health.

[34] O’Leary L, Cooper S-A, Hughes-McCormack L (2018). Early death and causes of death of people with intellectual disabilities: A systematic review. J Appl Res Intellect Disabil.

[35] Hughes-McCormack LA, Rydzewska E, Cooper S-A (2022). Rates, causes and predictors of all-cause and avoidable mortality in 163 686 children and young people with and without intellectual disabilities: a record linkage national cohort study. BMJ Open.

[36] Australian Government Department of Health and Aged Care (2023). National roadmap for improving the health of people with intellectual disability.

