# Supplementary material for: Life expectancy of people with intellectual disability: a retrospective cohort study from New South Wales, Australia
Source: BMJ Open. 2026 Feb 4;16(2):e110184. doi: 10.1136/bmjopen-2025-110184 (PMC12878197; doi:10.1136/bmjopen-2025-110184)
Supplement: Supplementary file 1 [file bmjopen-2025-110184.reviewer_comments.pdf]

PEER REVIEW HISTORY

BMJ Open publishes all reviews undertaken for accepted manuscripts. Reviewers are asked to complete a checklist review form and are provided with free text boxes to elaborate on their assessment. These free text comments are reproduced below.

ARTICLE DETAILS

Title (Provisional)

Life Expectancy of People with Intellectual Disability: A Retrospective Cohort Study from New South Wales, Australia

Authors

Srasuebkul, Preeyaporn; Trofimovs, Julian; Trollor, Julian

VERSION 1 - REVIEW

**Reviewer**	**1**
**Name**	**Bruland, Dirk**
**Affiliation**	**Bielefeld University of Applied Sciences**
**Date**	**30-Sep-2025**
**COI**	**None**

Thank you for the interesting contribution. The contribution deals with the life expectancy of people with intellectual disabilities. It compares this with the general population and takes a closer look at gender differences. Methods have been presented in a good comprehensible manner. The results are discussed with strenght and limitations and the results are well regarded. The STROBE reporting list have a lot of N/As. Maybe it is possible to write why there is no report possible for better understanding.

**Reviewer**	**2**
**Name**	**Wark, Stuart**
**Affiliation**	**University of New England, School of Rural Medicine**
**Date**	**03-Oct-2025**
**COI**	**None**

This paper reports on a retrospective cohort study of 100,089 NSW residents with intellectual disability in relation to life expectancy. This is a very important but under-researched area of public health, and has implications for both policy and practice. As noted by the authors, this manuscript represents the first life table analysis of its type in the

Australian context, and I thoroughly commend the authors for their focus on this project. I have no significant concerns with the manuscript in its current state.

**Reviewer**	**3**
**Name**	**Landes, Scott**
**Affiliation**	**Syracuse University, Department of Sociology and Aging Studies Institute**
**Date**	**13-Oct-2025**
**COI**	**None**

Well-written study on life expectancy for people with IDD in NSW. Extremely important topic. All my questions revolve around the sample. The authors state: “The linked data set comprised 20 individual administrative data sets from health, disability, and other services. Nine of these were used for cohort identification. Our intellectual disability cohort included all individuals who received disability services in NSW and had intellectual disability as their primary or secondary disability. It also included those with a recorded diagnosis of intellectual disability in health service data sets, as described by Reppermund et al. (20).”

I had to go read the Reppermund paper to get the information I needed, and even in doing that, could not get all the needed basics. First, I would present your methods so the reader does not have to go scrounging around the Reppermund article to find this info. It is annoying. Second, what were the 9 of 20 that were used, why these 9, and why were the 11 excluded? Third, what did “who received disability services in NSW” entail? Per my reading of Reppermund, this is a fairly expansive list of services, but it needs to be clearly stated what is/is not included. Fourth, who were those people who had a recorded dx but were not receiving services? Are they not the ones that you later state in the limitations: “Reliance on administrative data for cohort definition likely underestimates the intellectual disability population, as individuals not accessing services are excluded.” Obviously, you are of the mind that someone is being missed. Yet, it is not possible to tell from the presentation who this may be. In my mind, this is critical information needed to evaluate the validity of the study. Life tables are typically reserved for population data. It does not seem to me that you have population data, more so a select sample of some service recipients or people with an IDD indicator in their medical record, but this is my best guess as to who is included. I should not have to be guessing. This should be clearly stated – who is in and who is out. Doing so also may provide more insight to the potential direction of the bias. Finally, it will allow for delineation of whether life tables are the best strategy for this sample or not.

VERSION 1 - AUTHOR RESPONSE

	Response

Reviewer: 1 Dr. Dirk Bruland, Bielefeld University of Applied Sciences	
Thank you for the interesting contribution. The contribution deals with the life expectancy of people with intellectual disabilities. It compares this with the general population and takes a closer look at gender differences. Methods have been presented in a good comprehensible manner. The results are discussed with strength and limitations and the results are well regarded. The STROBE reporting list have a lot of N/As. Maybe it is possible to write why there is no report possible for better understanding.	We thank the reviewer for their positive feedback and thoughtful review of our manuscript. Regarding the STROBE checklist, we have now reviewed all N/A entries and added explanatory notes for each item that is not applicable to our study design. For example, items related to missing data is not applicable as our data used the National Death Index, hence, there is no missing mortality information. We hope these clarifications make our methodological choices more transparent.
Reviewer: 2 Dr. Stuart Wark, University of New England	
This paper reports on a retrospective cohort study of 100,089 NSW residents with intellectual disability in relation to life expectancy. This is a very important but under-researched area of public health and has implications for both policy and practice. As noted by the authors, this manuscript represents the first life table analysis of its type in the Australian context, and I thoroughly commend the authors for their focus on this project. I have no significant concerns with the manuscript in its current state.	We sincerely thank the reviewer for this encouraging message. We have further strengthened the manuscript in response to comments from other reviewers.
Reviewer: 3 Prof. Scott Landes, Syracuse University	
Well-written study on life expectancy for people with IDD in NSW. Extremely important topic. All my questions revolve around the sample. The authors state: "The linked data set comprised 20 individual administrative data sets from health, disability, and other services. Nine of these were used for cohort identification. Our intellectual disability cohort included all individuals who received disability services in NSW and had intellectual disability as their primary or secondary disability. It also included those with a recorded diagnosis of intellectual disability in health service data sets, as described by Reppermund et al. (20)."<br><br>I had to go read the Reppermund paper to get the information I needed, and even in	Firstly, we'd like to apologise to Prof. Landes for not providing more information about cohort formation in our first draft.<br><br>We have now amended the data source added more information about the datasets used for cohort formation. To address the missing 9/20 datasets information, we now add<br><br>"Nine of these datasets were available for cohort identification at the time of the study: Disability Service Minimum Dataset (DSMDS); Admitted Patient Data Collection; Mental Health Ambulatory Data; Emergency Department Data Collection; NSW Department of Education (students who received targeted specialist support in public schools; Statewide Disability Services, Corrective Services NSW; NSW Public

doing that, could not get all the needed basics. First, I would present your methods so the reader does not have to go scrounging around the Reppermund article to find this info. It is annoying.<br>Second, what were the 9 of 20 that were used, why these 9, and why were the 11 excluded?<br>Third, what did “who received disability services in NSW” entail? Per my reading of Reppermund, this is a fairly expansive list of services, but it needs to be clearly stated what is/is not included.	Guardian and NSW Ombudsman. These 9 datasets contained specific diagnosis codes or service provision flags necessary to identify individuals with intellectual disability.” (page 4, last paragraph and page 5 first paragraph)<br><br>We added a new paragraph to summarise why 11 datasets were not used to form our cohort. The new paragraph is shown below.<br><br>“The remaining 11 datasets (e.g. the NSW Registry of Births, Deaths and Marriages or Cancer Registry) were not used to identify individuals with intellectual disability as they do not contain these specific identifiers. Instead, these datasets were used to ascertain outcomes (i.e. mortality or cancer incidence) and establish covariates. Specifically, date of death information from the NSW Registry of Births, Deaths and Marriages (RBDM) and the National Death Index (NDI) datasets was used to determine the end of follow-up.” (page 5, second paragraph)<br><br>We have now identified what receiving disability services in NSW entailed by adding this text<br><br>“For this study, ‘appearing in a disability service dataset’ (e.g. DSMDS) was defined as accessing one or more state-funded supports such as supported accommodation, community participation or respite care.” (page 5, paragraph 3)
Fourth, who were those people who had a recorded dx but were not receiving services? Are they not the ones that you later state in the limitations: “Reliance on administrative data for cohort definition likely underestimates the intellectual disability population, as individuals not accessing services are excluded.” Obviously, you are of the mind that someone is being missed. Yet, it is not possible to tell from the presentation who this may be.	People who had a recorded diagnosis but were not receiving services were **included** in our study. We do not have people with intellectual disability who did not receive any disability or health care services (i.e. no recorded diagnosis). We have improved text clarity in the following manner:<br>1. Our revised Methods section now clearly states that the cohort was formed from individuals identified in either the disability service system OR the health system. The next now reads<br>“An individual was included in our study cohort if they were identified through at least one of the following pathways: 1) appearing in a disability service dataset with intellectual disability recorded as their primary or

	secondary disability, and 2) appearing in a health administrative dataset with a recorded diagnosis of intellectual disability." (page 5, paragraph 2)<br><br>2. Our revised limitations now clearly stated our cohort only excluded people with intellectual disability **who do not access any services** and it is likely to underestimate the intellectual disability population. The text now reads:<br>"However, a primary limitation is our reliance on administrative data for cohort formation. We acknowledge that our sample is an administrative cohort and not a complete population sample, as it does not capture the entire population of people with intellectual disability in NSW. This methodology likely underestimates the intellectual disability population, as individuals not accessing services, and those with intellectual disability who not identified as such are excluded." (page 8, paragraph 7 and page 9, paragraph 1)
In my mind, this is critical information needed to evaluate the validity of the study. Life tables are typically reserved for population data. It does not seem to me that you have population data, more so a select sample of some service recipients or people with an IDD indicator in their medical record, but this is my best guess as to who is included. I should not have to be guessing. This should be clearly stated – who is in and who is out. Doing so also may provide more insight to the potential direction of the bias. Finally, it will allow for delineation of whether life tables are the best strategy for this sample or not.	We have responded to the queries regarding 'who is in' and 'who is out' previously. This response in this section will be focused on the validity of life table methodology.<br><br>While we agree that our cohort is not a full capture of all individuals with intellectual disability in NSW, we have addressed that in our expanded limitations section (page 8, paragraph 7 and page 9, paragraph 1), including a detailed discussion of the potential direction of the bias.<br><br>Life table methodology is a standard analytical approach for examining mortality patterns in any well-defined cohort with complete follow-up, regardless of whether it represents a complete population. Examples in the literature include life tables constructed for:<br>- Disease registry cohorts (e.g., Life Expectancy in High-Grade Incidental Prostate Cancer Patients Versus Population-Based Controls According to Treatment Type, where the authors used SEER cancer registries for their cases and compared the life table with

	simulated age-matched controls (Di Bello et al 2025.))<br>- Insurance populations<br>- Health service users with specific conditions<br>- Occupational cohorts with specific exposures<br>Our study employs a period life table methodology, using age-specific mortality from 2001-2018 for individuals with intellectual disability identified through services in NSW. This cohort represents those visible to the health and disability-service systems – a population of direct policy relevance. We have complete mortality ascertainment through linkage to death registrations, and accurate person-time denominators, which are the essential requirements for valid life table construction.<br><br>The limitation is not in the methodology but in the generalisability, which we have explicitly addressed. Our life table provides valid estimates of mortality patterns of service-using individuals with intellectual disability, a critical population for health system planning.

Reference:

Di Bello F, Jannello LMI, Baudo A, de Angelis M, Siech C, Tian Z, Goyal JA, Creta M, Califano G, Celentano G, Acquati P, Saad F, Shariat SF, Carmignani L, de Cobelli O, Briganti A, Chun FKH, Longo N, Karakiewicz PI. Life Expectancy in High-Grade Incidental Prostate Cancer Patients Versus Population-Based Controls According to Treatment Type. Prostate. 2025 Feb;85(2):191-197. doi: 10.1002/pros.24816. Epub 2024 Oct 24. PMID: 39449158.

VERSION 2 - REVIEW

Reviewer 3

Name Landes, Scott

Affiliation Syracuse University, Department of Sociology and Aging Studies Institute

Date 01-Dec-2025

COI

Overall, edits strengthened the paper. The response to my last point was confusing. In essence it seemed to be - we don't have a population, but are using life tables to evaluate a cohort, that we are (mistakenly) referring to in the comments as a population. I am aware that life tables have been/are used for non population data. However, demographers such as myself, would push back that this is not good use of this method. My thought is, if possible, to provide a much clearer statement of the population studied in this paper. Per my read, it seems to be those receiving services in NSW. If that is the case, then it is simply a matter of defining the population as such. If you do not have this population, I am not totally against use of life tables, but think it would be best to acknowledge you do not have population data as well as the limitations of using life tables for non-population data.

VERSION 2 - AUTHOR RESPONSE

Comment	Response
Prof. Scott Landes, Syracuse University<br>Overall, edits strengthened the paper. The response to my last point was confusing. In essence it seemed to be - we don't have a population, but are using life tables to evaluate a cohort, that we are (mistakenly) referring to in the comments as a population. I am aware that life tables have been/are used for non population data. However, demographers such as myself, would push back that this is not good use of this method. My thought is, if possible, to provide a much clearer statement of the population studied in this paper. Per my read, it seems to be those receiving services in NSW. If that is the case, then it is simply a matter of defining the population as such. If you do not have this population, I am not totally against use of life tables, but think it would be best to acknowledge you do not have population data as well as the limitations of using life tables for non-population data.	We accept this point and appreciate the clarification regarding demographic terminology. We agree that defining our study group as the 'service user population' is the most accurate approach.<br><br>We have revised the manuscript to explicitly state that our data represents the 'population of people with intellectual disability receiving services in NSW'. We have removed references implying this is a total population. We also added a statement acknowledging the limitations of using life tables for this specific service population.<br><br>We have revised the text in the manuscript as follows:
	We updated the **'Strengths and Limitations'** section (page 2). We modified one heading and replaced the limitation on group comparisons with a new point titled 'Generalisability of service data'. The text now reads:<br><br>- **Service-user specific estimates**: Our approach directly yields life expectancy estimates for **the service user population with intellectual disability** (with or without Down syndrome). This provides a precise baseline for future calculations of health

	metrics, including the burden of disease in people with intellectual disability, which cannot be derived from general population life tables.<br><br>- **Generalisability of service data:** We derived these life tables from administrative service data, consequently, findings reflect the mortality experience of the service user population rather than the total population of people with intellectual disability.
	We revised the **'Methods'** section to explicitly define the sample as the 'service user population' in NSW. We also changed the term 'subpopulation' to 'subgroup' to avoid confusion regarding demographic definitions (page 5, paragraph 5). The text now reads:<br><br>Our study sample comprised 100,089 service users with intellectual disability in NSW, spanning birth through December 31, 2018. We excluded individuals who had died before January 1, 2001, and those born after December 31, 2018. The sample was stratified by sex and Down syndrome status. We identified a subgroup of individuals with Down syndrome using diagnostic codes from the linked datasets (ICD-9 code 758.0, ICD-10 code Q90, or ICD-10-AM code U88.2) or any flag of Down syndrome from disability service datasets.<br><br>We clarified the interpretation of the results in the **'Life expectancy estimation'** section (page 6). The revised text now reads:<br><br>Finally, we derived life expectancy at birth and remaining life expectancy at selected ages (e.g., 20, 40, 60) directly from these calculations. Given that the latest birth year included in our cohort was 2018, life expectancy at birth derived from these period life tables can be interpreted as the life expectancy for individuals with intellectual disability who were born in 2018, **assuming they experience the same age-specific mortality rates as observed in our study cohort throughout their lives.** We calculated 95% confidence intervals for life expectancy at birth using the parametric bootstrap method, assuming a Poisson

	distribution of deaths in each group with 1000 replications.
	We have further clarified our service user cohort in the **'Results'** section on page 6, paragraph 5. The revised text now reads:<br><br>Detailed cohort characteristics have been reported previously. [20] Briefly, the study included 100,089 **service users with intellectual disability**, of whom 34% were female, with a median age at cohort entry of 3 years (interquartile range, 0-19). Down syndrome was present in 4% of the cohort, and 8911 deaths occurred during the study period.
	Regarding the reviewer's point on the use of the life table for non-population data. We agree with the reviewer that it is essential to acknowledge the limitations of using life tables for non-population data. The **'Discussion'** and **'Limitations'** sections explicitly addresses this methodological validity. The revised text now reads:<br><br>**Discussion**<br><br>Page 7, last paragraph<br><br>This study provides contemporary and robust estimates of life expectancy for **the service user population** with intellectual disability in New South Wales (NSW), Australia. We found a substantial 16-year reduction in life expectancy at birth (67 years) compared to the general NSW population, with particularly pronounced disparities for individuals with Down syndrome (54.55 years). While males and females with intellectual disability had similar life expectancy, the disparity with the general population was greater for females [29]. The life expectancy gap diminished with age across all subgroups.<br><br>**Limitations**<br><br>Page 8, paragraph 5<br><br>This study has several notable strengths. We utilised a large population-based linked administrative dataset from NSW, Australia's most populous state representing approximately one-third of the national

	population, providing robust and contemporary estimates. The application of formal life table methodology ensured transparency and direct comparable estimates. Our approach enabled detailed stratification by sex and Down syndrome status, yielding **service-user** population-specific life expectancy estimates crucial for health planning and policy development.<br><br>Page 9, 1st paragraph<br><br>Despite this limitation in generalisability, the use of life tables remains the gold standard and most statistically robust method for calculating and presenting age-specific mortality rates and life expectancy for any defined longitudinal cohort. A core objective of this study was to comprehensively analyse life expectancy for people with intellectual disability to inform health equity and service planning. This objective required the generation of robust, cohort-specific estimates, as applying general population tables would be inappropriate and misrepresent the true mortality burden for this group.<br><br>**Conclusion**<br><br>Page 9, last paragraph<br><br>This study demonstrates significant life expectancy disparities for **service users** with intellectual disability in NSW, with a 16-year reduction at birth and additional 12-year reduction for Down syndrome.
